# Morphological Control of Supported ZnO Nanosheet Arrays and Their Application in Photodegradation of Organic Pollutants

**DOI:** 10.3390/nano13030443

**Published:** 2023-01-21

**Authors:** Jun Wang, Bo Gao, Dongliang Liu, Lin Cheng, Yu Zhang, Dingze Lu, Huawa Yu, Aimin Chen, Shun Yuan, Kaijia Chen, Shiguang Shang

**Affiliations:** 1 School of Science, Xi’an Polytechnic University, 19 Jinhua South Road, Xi’an 710048, China; 2 School of Science, Xi’an Jiaotong University, 28 Xianning Road, Xi’an 710049, China; 3 School of Electronic Engineering, Xi’an University of Posts and Telecommunications, 1 Chang’an West St. Xi’an 710121, China

**Keywords:** supported nanostructured photocatalyst, ZnO nanosheet array, morphological control, one-step hydrothermal fabrication

## Abstract

Supported nanostructured photocatalysis is considered to be a sustainable and promising method for water pollution photodegradation applications due to its fascinating features, including a high surface area, stability against aggregation, and easy handling and recovery. However, the preparation and morphological control of the supported nanostructured photocatalyst remains a challenge. Herein, a one-step hydrothermal method is proposed to fabricate the supported vertically aligned ZnO nanosheet arrays based on aluminum foil. The morphologically controlled growth of the supported ZnO nanosheet arrays on a large scale was achieved, and the effects of hydrothermal temperature on morphologic, structural, optical, and photocatalytic properties were observed. The results reveal that the surface area and thickness of the nanosheet increase simultaneously with the increase in the hydrothermal temperature. The increase in the surface area enhances the photocatalytic activity by providing more active sites, while the increase in the thickness reduces the charge transfer and thus decreases the photocatalytic activity. The influence competition between the area increasing and thickness increasing of the ZnO nanosheet results in the nonlinear dependence between photocatalytic activity and hydrothermal temperature. By optimizing the hydrothermal growth temperature, as fabricated and supported ZnO nanosheet arrays grown at 110 °C have struck a balance between the increase in surface area and thickness, it exhibits efficient photodegradation, facile fabrication, high recyclability, and improved durability. The RhB photodegradation efficiency of optimized and grown ZnO nanosheet arrays increased by more than four times that of the unoptimized structure. With 10 cm^2^ of as-fabricated ZnO nanosheet arrays, the degradation ratio of 10 mg/L MO, MB, OFL, and NOR was 85%, 51%, 58%, and 71% under UV irradiation (365 nm, 20 mW/cm^2^) for 60 min. All the target pollutant solutions were almost completely degraded under UV irradiation for 180 min. This work offers a facile way for the fabrication and morphological control of the supported nanostructured photocatalyst with excellent photodegradation properties and has significant implications in the practical application of the supported nanostructured photocatalyst for water pollution photodegradation.

## 1. Introduction

Over the past decade, water pollution caused by organic dye and antibiotic residues has become increasingly serious due to its widespread use in various industries and modern medicine [1]. As an efficient, economical, and green water pollution treatment, photocatalytic degradation can mineralize organic dye and antibiotic residues into H_2_O, CO_2_, and mineral acids by utilizing solar light [2]. Due to the nontoxic nature, low cost, and high activity [3], zinc oxide (ZnO) nanoparticles for photodegradation in wastewater have been extensively investigated, such as ZnO nanoflowers [4], ZnO nanoflakes [5,6], ZnO nanobelts [7], etc. They exhibit excellent catalytic performance for water pollution photodegradation owing to numerous advantages, including high surface-to-volume ratio, porous structures, as well as enhanced light harvesting [8]. However, ZnO nanoparticles have the tendency to aggregate and require a post-treatment process for the catalysts’ separation and reuse [1]. This strategy increases operating costs, reduces the photocatalyst’s reusability, and makes water pollution photodegradation application at an industrial scale unsustainable. To address the aforementioned problem of ZnO nanoparticles, extensive research has been carried out in recent years on various supported ZnO morphologies and arrays, including ZnO nanowire arrays [9,10], ZnO nanorod arrays [11,12], and ZnO nanosheet arrays [13,14,15,16].

ZnO nanosheet arrays are regarded as promising two-dimensional supported ZnO nanostructures and have attracted enormous attention due to large specific surface areas and nanoscale thickness [16]. Compared with one-dimensional supported ZnO structures such as ZnO nanowire or nanorod arrays, ZnO nanosheet arrays have higher specific surface areas and provide a larger number of active sites to achieve better performance for photodegradation in wastewater. Meanwhile, ZnO nanosheet arrays provide a comparably shorter distance for the photo-induced charge to rapidly reach the surface of the nanosheet, leading to enhanced charge transfer and electron-hole pairs separation. In the past few years, a tremendous effort has been made to fabricate supported ZnO nanosheet arrays. Wang et al. [13] reported a type of nanosheet-based ZnO thin film prepared by the hydrothermal oxidation of Zn foil in an alkaline aqueous solution. Sun et al. [14] developed a seed-assisted hydrothermal growth method to fabricate nest-like ZnO arrays on glass substrates. Chen et al. [15] proposed annealing a sheet-like precursor coated on Zn foils to synthesize porous ZnO nanosheet arrays and applied this to the degradation of methyl orange. Banerjee et al. [16] demonstrated the synthesis of the ZnO nanosheet array by using the room temperature high-power sonochemical method of synthesis. It should be pointed out that although ZnO nanosheet arrays have been successfully fabricated by various methods, there are rare reports on the precise morphology control of supported ZnO nanosheet arrays. It is well known that the morphologies of ZnO nanostructures are confessed to having great effects on their photocatalytic properties and corresponding potential applications. Thus, the development of convenient and facile strategies to control the morphology-supported ZnO nanosheet arrays is particularly critical.

In this study, supported ZnO nanosheet arrays based on aluminum foil were facilely fabricated and morphologically controlled using a one-step hydrothermal method. By means of various characterization methods, the hydrothermal temperature effects on the morphologic, structural, and optical properties were investigated. The increase in the surface area and thickness of ZnO nanosheet arrays with the increase in the hydrothermal temperature effect was analyzed. The corresponding growth mechanisms and the influence competition between the area increasing and thickness increasing of ZnO nanosheets were discussed. Photocatalytic activities that degrade cationic, anionic dyes and fluoroquinolones antibiotics under ultraviolet illumination were evaluated, including rhodamine b (RhB), methyl orange (MO), methylene blue (MB), ofloxacin (OFL) and norfloxacin (NOR). In addition, photocatalytic cyclical stability and durability as supported nanostructured photocatalysts were studied. This work attempts to demonstrate the hydrothermal temperature effects on the area and thickness simultaneously and the corresponding influence competition mechanisms, which effectively regulate photogenerated electron-hole production and the charge transfer process, thus significantly affecting the photocatalytic activity of ZnO nanosheet arrays. This strategy is not limited to the field of photocatalysis but also involves solar cells, electronics, optoelectronics, and other fields.

## 2. Materials and Methods

### 2.1. Materials

Hexamethylenetetramine (HMTA) and zinc nitrate hexahydrate (Zn(NO_3_)_2_·6 H_2_O) were purchased from the Shanghai Chemical Reagent Company (Shanghai, China). Organic dyes and antibiotics in our experiments were obtained from Shanghai Aladdin Biochemical Technology Company Limited (Shanghai, China). These reagents were of analytical grade and used without further purification. The aluminum foils (0.3 mm thickness) used in this experiment were purchased from Suzhou Metal Material Company (Suzhou, China). Deionized water (resistance > 18 MΩ cm^−1^) was obtained from the Millipore water purification system.

### 2.2. One-Step Hydrothermal Preparation of Supported ZnO Nanosheet Arrays

First, the aluminum foils were cleaned with an ultrasonic cleaner to remove contaminants. A growth solution of 0.025 mol/L was prepared with a molar ratio of 1:1 hexamethylenetetramine and zinc nitrate. Then, the aluminum foils were placed vertically in the inner pot of the hydrothermal kettles for 2h. The kettles were held at different hydrothermal temperatures, at 80 °C, 95 °C, 110 °C, 125 °C, and 140 °C, respectively. At last, ZnO nanosheet arrays were cleaned with deionized water and dried in the air at room temperature.

### 2.3. Characterization of Supported ZnO Nanosheet Arrays

The surface morphologies of the fabricated samples were analyzed using scanning electron microscopy (SEM, FEI Quanta 450, FEI Company, Hillsboro, OR, USA) with an acceleration voltage of 20 kV. The specific surface areas of the fabricated samples were measured using a nitrogen gas adsorption/desorption surface area tester (JWGB BK400, Beijing, China) at 77 K and were calculated by the multipoint Brunauer–Emmett–Teller (BET) method. The crystallinity and crystalline phases were studied by X-ray diffraction (XRD, BUKER D8 Advance, Karlsruhe, Germany) with Cu Kα radiation (λ = 1.5406 Å) in the 2-theta range between 10° and 60° with a scanning rate of 0.02°/min. The composition and elemental states of various ions were identified by X-ray photoelectron spectroscopy (XPS, THERMO Escalab 250Xi, ThermoFisher Scientific, Waltham, MA, USA) equipped with a monochromatized Al–Kα X-ray source. The values of the binding energies were calibrated according to the energy of standard C 1s peak (284.8 eV). The Avantage software was used to process and analyze XPS spectra. The optical properties of the fabricated samples were recorded by ultraviolet and visible reflection spectroscopy (UV-Vis reflection, IDEAOPTICS Nova, Shanghai, China) equipped with an integrating sphere with assembly and 100% commercial BaSO_4_ as the reflectance sample. The photo-induced charge carrier separation and recombination processes were analyzed using photoluminescence spectroscopy (PL, HORIBA Fluoromax4, Piscataway, NJ, USA) at room temperature. PL spectroscopy in the 350 to 600 nm range was obtained with the excitation at 325 nm. Both the excitation and emission slit widths were set at 2 nm.

### 2.4. Photodegradation Activity Measurement of Supported ZnO Nanosheet Arrays

In order to demonstrate the photodegradation activity of fabricated and supported nanosheet arrays, RhB was used as the target degradation. In the photodegradation experiment, five pieces of 1 × 2 cm supported nanosheet arrays were first placed in the 50 mL pollutants solution (10 mg/L) for 1 h under dark conditions to ensure that the adsorption–desorption balance between ZnO nanosheet arrays and pollutants solution was reached. Then, the photodegradation of pollutant solutions was carried out under the UV irradiation of a 100 W mercury lamp (365 nm, 20 mW/cm^2^) for 3 h, and a 0.5 mL degraded solution as the samples were taken every half an hour. Cold water circulating and magnetic stirring were maintained during the above process. At last, the adsorption spectra of the samples were recorded with a fiber optic spectrometer to estimate the photodegradation activity of fabricated ZnO nanosheet arrays.

### 2.5. Photocatalytic Cyclical Stability and Durability of Supported ZnO Nanosheet Arrays

The photocatalytic cyclical stability measurement was performed by recycling the ZnO nanosheet arrays 5 times. In our experiment, the reused samples were simply rinsed three times with water and dried at 80 °C before again repeating the same photodegradation process. Durability measurements were performed with high-intensity ultrasound (100 W, 30 min) treatment to simulate the destruction of the sample. As a comparison, silicon-based ZnO nanosheet arrays were prepared using the two-step seed crystal growth method in the literature [17], and durability measurements were also performed.

### 2.6. Various Pollutants Photodegradation of ZnO Nanosheet Arrays

Four different pollutants were used as the target degradation, including MO, MB, OFL, and NOR. For the convenience of the study, all the concentrations of the pollutants solution in this work were chosen to be 10 mg/L. Other details were consistent with the RhB photodegradation.

## 3. Results and Discussion

### 3.1. The Morphology of Supported ZnO Nanosheet Arrays

**Figure 1:**
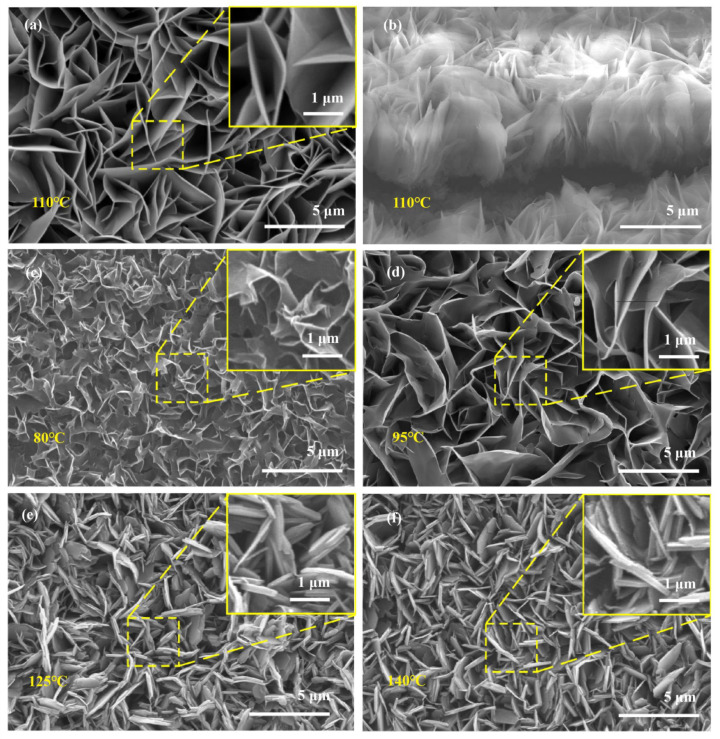
SEM images of supported nanosheet with different hydrothermal temperatures (**a**) Top-view SEM image of nanosheet arrays grown at 110 °C (**b**) Oblique-view SEM image of nanosheet arrays grown at 110 °C (**c**) 80 °C (**d**) 95 °C (**e**) 125 °C, and (**f**) 140 °C. The insets are the high magnification SEM image of the corresponding arrays.

To demonstrate the morphologic features of as-fabricated arrays and evaluate hydrothermal temperature effects, the surface morphology analysis of supported ZnO nanosheet arrays grown at various hydrothermal temperatures was carried out through the FE-SEM technique, and the results are represented in Figure 1. Figure 1a,b show the top-view and oblique-view SEM diagrams of the supported nanosheet arrays grown at 110 °C, respectively. The inset figures show the enlarged FE-SEM images of the corresponding supported ZnO nanosheet arrays. As illustrated in Figure 1a, as-prepared and supported nanosheet arrays are covered with dense and uniform sheet-like nanostructures. From the inset of Figure 1a, these sheet-like nanostructures are on a micron-scale, and the thickness is at the nanoscale. These nanosheets are widespread over the whole substrate, as shown in the large-scale SEM images (Figure S1) at low magnifications. As pointed out in the literature [16], these two-dimensional planar nanosheets can provide more catalytically active sites at the edge and plane of nanosheets along with the facile separation and transport of photogenerated electron–hole pairs, leading to the improvement of photocatalytic reaction kinetics. It was further found that these sheet-like nanostructures are vertically grown on the substrate, as shown in Figure 1b. Compared to the horizontally aligned nanosheet arrays, the vertically aligned nanosheet array could avoid the overlap between the nanosheets and have more potential to enhance the catalytically active redox reactions.

Figure 1c–f shows the hydrothermal temperature effects on the morphology of supported ZnO nanosheet arrays grown at 80 °C, 95 °C, 125 °C, and 140 °C, respectively. As illustrated in Figure 1c–f, the fabricated arrays were grown at various hydrothermal temperatures and show similar nanosheet array morphological characteristics. However, the influences of the hydrothermal temperature on the size and thickness of the supported nanosheet are also clearly observed from the inset of Figure 1c–f. At 80 °C, the supported nanosheet begins to form but is not complete (Figure 1c). At 95 °C, the fabricated array exhibits significant sheet-like characteristics, and the size of the nanosheet is remarkably larger than 80 °C (Figure 1d). At 125 °C and 140 °C, the supported nanosheet arrays become further thickened and even stacked together (Figure 1e,f).

In order to quantitatively reveal the morphological changes of fabricated ZnO nanosheet arrays with different hydrothermal temperatures, the specific surface areas of ZnO nanosheet arrays and the nanosheets’ thickness variation analysis were carried out, and the results are listed in Table 1. The specific surface areas of the ZnO nanosheet arrays were evaluated by a multipoint BET method, and the corresponding N_2_ adsorption/desorption isotherm at 77 K of the samples grown at different hydrothermal temperatures are shown in Figure S2. From Figure S2 and the subsequent description, the specific surface areas are shown to simultaneously increase alongside the increase in hydrothermal temperatures. These results indicate that the overall adsorption abilities of the ZnO nanosheet array for pollutants photodegradation is enhanced and may improve photodegradation activity. On the other hand, the thicknesses of the supported nanosheet arrays grown at various hydrothermal temperatures were comprehensively measured with imageJ software, and the thicknesses data were fitted with the Gaussian model. The statistical fitting curves and methodology details are shown in Figure S3 and the subsequent description. From Figure S3 and the subsequent description, the average thicknesses of the nanosheets are shown as gradually becoming larger with the increase in hydrothermal temperature. The average thickness of ZnO nanosheet arrays grown at 140 °C was four times thicker than that of ZnO nanosheet arrays grown at 80 °C. It can be predicted that these variations of the zno nanosheet arrays’ specific surface area and nanosheets’ thickness with the increase in hydrothermal temperature may greatly affect the photodegradation performance of ZnO nanosheet arrays.

### 3.2. Structural Characteristics of Supported ZnO Nanosheet Arrays

**Figure 2:**
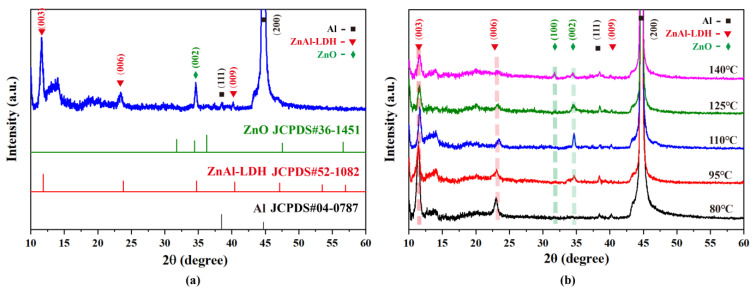
(**a**) XRD spectrum of supported nanosheet arrays grown at 110 °C (**b**) Comparative XRD studies of supported nanosheet arrays grown at various hydrothermal temperatures.

**Figure 3:**
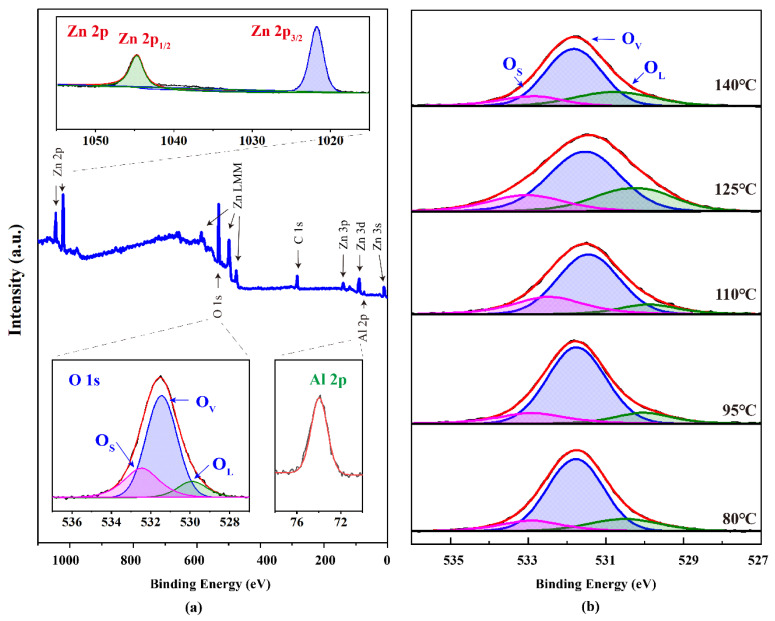
(**a**) XPS spectrum of supported ZnO nanosheet arrays grown at 110 °C. Major spectral lines and auger lines are labelled. The inset is the narrow scan corresponding to Zn 2p, O 1s, and Al 2p (**b**) Variations in O 1s narrow scan with different hydrothermal temperatures.

Figure 2 shows the XRD patterns of fabricated nanosheet arrays grown at 110 °C and other hydrothermal temperatures. As depicted in Figure 3a, the intense diffraction peaks represented by black square at 38.4° and 44.7°correspond to the (111) and (200) planes of the Aluminum substrate (JCPDS 04-0877). The diffraction peak represented by the olive diamond at 34.5°corresponds to the (002) plane of ZnO (JCPDS 36-1451), confirming the formation of ZnO. Meanwhile, no other orientated peak corresponding to the crystallographic plane of ZnO was observed, suggesting that ZnO grew preferentially along the C axis and was perpendicular to the substrate. The additional diffraction peak represented by the red inverted triangle at 11.6°, 23.6°, and 39.4° corresponds to the (003), (006), and (009) planes of ZnAl-LDH (JCPDS 52-1082), confirming the formation ZnAl-LDH at the interface of the ZnO nanosheet arrays and Aluminum substrate, as generally reported in the literature [17]. Comparative XRD studies of supported nanosheet arrays grown at various hydrothermal temperatures in Figure 3b reveal the hydrothermal temperature effects on the structural characteristics of supported ZnO nanosheet arrays. It is clear that with increasing hydrothermal temperature, the intensities of the diffraction peaks corresponding to ZnAl-LDH continuously decrease while the intensities of diffraction peaks corresponding to ZnO increase, indicating that a higher hydrothermal temperature is conducive to the fast formation of ZnO nanosheet arrays. Moreover, the intensity variations of diffraction peaks corresponding to (002) and (100) for ZnO suggest the growth process of ZnO nanosheet arrays. When the hydrothermal temperatures increase from 80 °C to 110 °C, the (002) peak’ intensity gradually increases, suggesting the nanosheet radial growth that is perpendicular to the substrate and resulting in the increment of the nanosheet area. When the hydrothermal temperatures increase from 110 °C to 140 °C, the (100) peak’ intensity becomes obvious, suggesting that the nanosheet axial growth was parallel to the substrate and resulting in the increment of nanosheet thickness. These results from XRD analysis are in agreement with the aforementioned morphological analysis.

The composition and elemental states of various ions present in as-fabricated supported ZnO nanosheet arrays were identified by XPS analysis. Figure 3a demonstrates the XPS spectrum of supported ZnO nanosheet arrays grown at 110 °C. Major spectral lines and auger lines in the survey XPS spectrum (Figure 3a) are labeled [18,19], which indicates that the sample was composed of Zn, O, and Al. A weak C 1s emission peak can be observed in the spectrum, which results from the sample holder and adventitious carbon present on the essential sample surfaces exposed to the environmental air. No peaks of other elements can be observed. From the wide scan of the XPS spectrum in Figure 3a, it can be seen that the XPS spectrum exhibits two apparent peaks related to Zn 2p, and O 1s, indicating that the main components of the nanosheet array are Zn and O elements. Meanwhile, the delicate peak appearing at the 74 eV related to Al 2p suggests the presence of trace Al elements. To provide more details of the composition and elemental states, the narrow scans of these characteristic peaks were analyzed by peak fitting and separation. For the narrow scan of Zn 2p, Zn 2p_1/2_ and Zn 2p_3/2_ peaks are located at 1044.7 and 1021.5 eV. The spin-orbit splitting between the two peaks is 23.2 eV, indicating a Zn^2+^ oxidation state in the ZnO wurzite lattice [20]. For the narrow scan of Al 2p, the symmetrical peak of Al 2p is located around 74.0 eV, suggesting that Al^3+^ ions formed Al–O bonds. Furthermore, as shown in the narrow scan of O 1s, three fitted peaks at 532.3 eV (O_S_), 531.3 eV (O_V_), and 529.7 eV (O_L_) are found and relate to oxygen adsorption and loosely bound oxygen (OH), oxygen vacancies in ZnO and Al–O bonds, and the lattice oxygen in the ZnO wurzite, respectively [21]. Figure 3b represents variations of the O 1s narrow scan with different hydrothermal temperatures. An increment in the proportion of O_L_-fitted peaks can be clearly seen with increasing hydrothermal temperatures and indicates the formation of ZnO wurzite. In addition, the proportion of the O_L_-fitted peak grown at 110 °C achieved a maximum value, suggesting the strongest adsorption of oxygen and OH (H_2_O) on the surface. In addition, as can be seen from Figure S4, the binding energy of Al 2p and Zn 2p is almost unchanged with increasing hydrothermal temperature. However, the ratio of them becomes smaller, which indicates that the proportion of zinc element gradually increased, while the proportion of the aluminum element gradually decreased.

### 3.3. Growth and Hydrothermal Temperature Effect Mechanism of Supported ZnO Nanosheet Array

It is well known that ZnO often crystallizes as a wurzite structure with two polar charged surface planes: the chemically active ${\mathrm{Zn}}^{2+}$ terminated (0001) plane and the inert O^2−^ terminated (000-1) surface. In the literature, the growth mechanism of the ZnO nanorod array [20] is reported; the ${\mathrm{OH}}^{-}$ ion provided by the HMTA is attracted to the terminated (0001) plane and combines with ${\mathrm{Zn}}^{2+}$ to form the $\mathrm{Zn}{\left(\mathrm{OH}\right)}_{4}^{2-}$ ion. It eventually grows into ZnO and H_2_O and results in the growth of ZnO nanorods. However, the growth mechanism of ZnO nanosheet arrays becomes different in the presence of the Al substrate. As known, HMTA produces ${\mathrm{OH}}^{-}$ ions and creates a hydrolysis process. Under alkaline conditions, Al can be oxidized and dissolved into the solution to form $\mathrm{Al}{\left(\mathrm{OH}\right)}_{4}^{-}$. In the vicinity of the substrate, due to the high concentration of $\mathrm{Al}{\left(\mathrm{OH}\right)}_{4}^{-}$ and $\mathrm{Zn}{\left(\mathrm{OH}\right)}_{4}^{2-}$, the proportion of zinc and aluminum are met, and thus, the nucleation and growth of ZnAL LDH are generated on the Al substrate. For a certain distance from the substrate, the certain proportion of zinc and aluminum is no longer met due to the decrease in the concentration of aluminum ions and ZnAl LDH, which is no longer formed. Instead, ZnO starts to nucleate. Because $\mathrm{Al}{\left(\mathrm{OH}\right)}_{4}^{-}$ could presumably bind to the positively charged ${\mathrm{Zn}}^{2+}$ terminated (0001) surface and inhibit the growth along the [0001] direction, 2D radial growth is achieved to form vertically aligned ZnO nanosheet arrays.

Considering the effect of hydrothermal temperature on the reaction kinetics and ${\mathrm{OH}}^{-}$ ions released from HMTA, the possible roles that hydrothermal temperature play on the morphology of the supported nanosheet array are discussed in two aspects. As the hydrothermal temperature increases, the supersaturation level in the growth solution is higher, resulting in the increase of nucleation and growth rates and, thus, ZnO nanosheet rapid growth. On the other hand, the increase in hydrothermal temperature benefits the ${\mathrm{OH}}^{-}$ ions released from HMTA. When the hydrothermal temperature is low, ${\mathrm{OH}}^{-}$ ion release from HMTA is slow, and not enough ${\mathrm{OH}}^{-}$ ions are released for the nucleation of ZnO. In this case, the growth of ZnO is restrained and incomplete, as shown in Figure 1c. As the hydrothermal temperature increases, the release rates of ${\mathrm{OH}}^{-}$ ions from HMTA are speeded up, and the concentration of ${\mathrm{OH}}^{-}$ ions produced by HMTA rise, and more ZnO nucleation begins to form. Owing to the Al substrate hydrolysis under alkaline conditions ${\mathrm{Al}}^{3+}$ combines with ${\mathrm{OH}}^{-}$ to generate $\mathrm{Al}{\left(\mathrm{OH}\right)}_{4}^{-}$ bonds that are stronger to the ZnO (0001) surface, resulting in the growth of a thinner nanosheet, as shown in Figure 1d. When the hydrothermal temperature further increases, HMTA dissolves rapidly and generates sufficient ${\mathrm{OH}}^{-}$. Due to the ${\mathrm{Zn}}^{2+}$ rich solution, the formations of $\mathrm{Zn}{\left(\mathrm{OH}\right)}_{4}^{2-}$ become faster. In contrast, limited by the rate of Al dissolution, aluminum hydroxide formation becomes inadequate. As the coverage rate of the $\mathrm{Al}{\left(\mathrm{OH}\right)}_{4}^{-}$ ions on the (0001) surface decreases, more and more $\mathrm{Zn}{\left(\mathrm{OH}\right)}_{4}^{2-}$ are attracted to the (0001) plane and grow along the [0001] direction, resulting in the increment of nanosheet thickness, as shown in Figure 1e–f. The growth processes and hydrothermal temperature effects of the ZnO nanosheet arrays are consistent with the XRD analysis in Figure 3b.

### 3.4. Optical Properties of Supported Nanosheet Arrays

**Figure 4:**
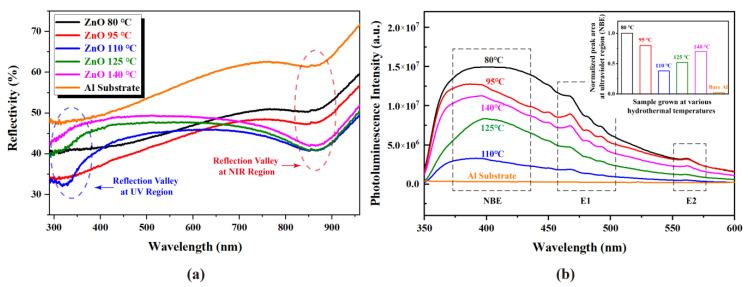
(**a**) UV-Vis reflection spectra (**b**) Photoluminescence spectra of bare Al substrate and fabricated nanosheet arrays grown at various hydrothermal temperatures. The inset of Figure 4b are normalized NBE’s integral areas.

Figure 4 demonstrates how the UV-Vis reflection and PL spectrum of bare Al substrate and fabricated nanosheet arrays are grown at various hydrothermal temperatures. From Figure 4a, it can be seen that compared with the bare Al substrate, as-fabricated ZnO nanosheet arrays have almost lower reflectance over the spectral range of 300–900 nm, indicating better absorptions of ZnO nanosheet arrays. In particular, the reflection spectrum of the ZnO nanosheet array grown at 110 °C exhibits two deep reflection valleys near 325 nm and 850 nm. These two reflection valleys are located in different spectral regions, and the physical mechanism of their generation is also different. The reflection valleys at 850 nm in the near-infrared region are mainly due to the inter-band transition of Al [22], which can also be obviously observed in other ZnO nanosheet arrays and bare Al substrates. As a comparison, the reflection valleys at 325 nm in the ultraviolet region only appear in the reflection spectrum of the nanosheet array grown at 110 °C, which is attributed to the high ultraviolet light absorptions of the ZnO and ZnAl LDH interlayer.

**Figure 5:**
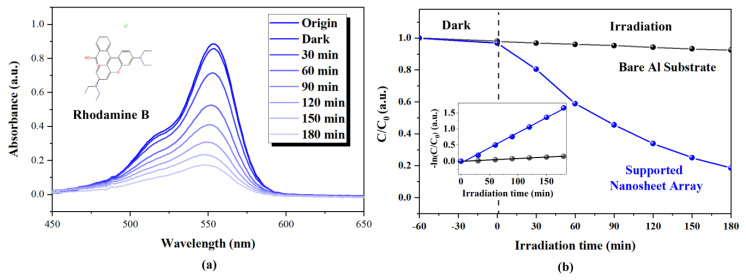
(**a**) Changes in UV-Vis absorption spectrum of photodegraded RhB solution with supported nanosheet arrays grown at 110 °C as a function of irradiation time (**b**) RhB concentration changes over bare Al substrate and supported nanosheet arrays as a function of irradiation time. The inset is the pseudo-first-order kinetic fitting plot of photodegradation.

In order to investigate the photo-induced charge carrier separation and recombination processes and other important information, such as surface defects and oxygen vacancies, room temperature PL measurements of fabricated nanosheet arrays grown at various hydrothermal temperatures with 325 nm excitation were carried out in our experiment. As demonstrated in Figure 4b, the bare Al substrate has almost no photoluminescence effect. On the contrary, the fabricated nanosheet arrays grown at various hydrothermal temperatures almost exhibit a strong UV emission band of around 390 nm and two weak visible light emission bands at about 460 nm and 560 nm, which are described as near-band-edge (NBE), E1 and E2 in Figure 4b, respectively. As demonstrated in the literature [23], the observed UV emission band can be attributed to the direct recombination of the conductance band electrons to the valence band holes, while the visible light emission bands are associated with the electron transfer from different defect states of ZnO such as oxygen vacancies and Zn interstitials. It can further be seen in Figure 5b that the intensity of the emission peak of the supported nanosheet array decreases first and then increases with the increasing hydrothermal temperature. The inset of Figure 4b shows that the NBE’s integral area of nanosheet arrays grown at 110 °C is the smallest. As is well known, in general, the lower the PL intensity, the lower the recombination rate of photo-induced electron–hole pairs, and the higher the photocatalytic activity of photocatalysts [23]. Therefore, ZnO nanosheet arrays grown at 110 °C are expected to achieve excellent photocatalytic performance for water pollution photodegradation applications.

### 3.5. Photocatalytic Properties of Supported ZnO Nanosheet Array Grown at 110 °C

**Figure 6:**
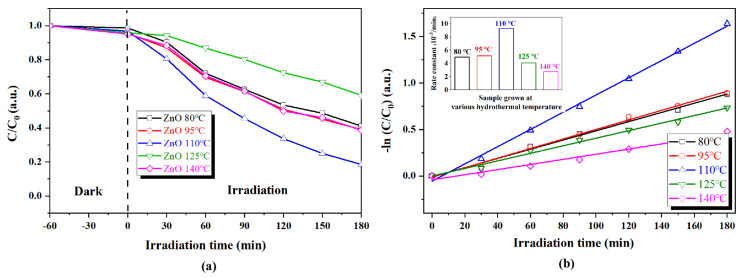
(**a**) RhB concentration changes over nanosheet arrays grown at various hydrothermal temperatures as a function of irradiation time (**b**) Pseudo-first-order kinetic fitting plots. The inset is photodegradation rate constant comparison.

Figure 5 shows the photodegradation of RhB dye pollution with supported nanosheet arrays grown at 110 °C. As shown in Figure 5a, the characteristic absorption peak of the RhB dye molecule became significantly weaker as photodegradation time went on, indicating the efficient photodegradation of RhB dye pollutions with supported nanosheet arrays. Figure 6b depicts a comparison of the photodegradation of the RhB dye molecule with the bare Al substrate and the supported nanosheet arrays grown at 110 °C. By using the bare Al substrate, the dye molecules slightly degraded (8.6%) under 180 min UV light irradiation due to light-induced self-degradation [24]. In contrast, using supported nanosheet arrays grown at 110 °C, the dye molecules degraded to 81.2% of their original concentration. It can be further found from the inset of Figure 5b that the photodegradation of RhB using supported nanosheet arrays grown at 110 °C follows the pseudo-first-order of kinetics. The pseudo-first-order rate constants k of the supported nanosheet arrays grown at 110 °C reach 0.00935 min^−1^, which is 10 times higher than that of RhB’s self-degradation. According to the photocatalytic results, the mechanism for the degradation of the RhB solution by ZnO nanosheet arrays could be explained. Upon the UV light irradiation, the conduction-band electrons and valence-band holes were generated on the surfaces of the ZnO nanosheet. The electrons can activate molecular oxygen to form superoxide radical anion $O_{2}^{•-}$, and the holes react with water to form highly reactive hydroxyl radicals ${}^{•}OH$. Both $O_{2}^{•-}$ and ${}^{•}OH$ have strong oxidative abilities, which are able to degrade RhB into CO_2_, H_2_O, and other intermediates.

In order to evaluate the hydrothermal temperature effect on the photodegradation activity of fabricated supported nanosheet arrays, the photodegradation experiment of RhB dye pollutions with supported ZnO nanosheet arrays grown at other hydrothermal temperatures were carried out, and the results are shown in Figure 6. The changes in the UV–Vis absorption spectrum of the photodegraded RhB solution as a function of irradiation time with supported nanosheet arrays grown at 80 °C, 95 °C, 125 °C, and 140 °C are shown in Figure S5. It can be clearly observed from Figure 6a that the photodegradation activities of ZnO nanosheet arrays depended strongly upon hydrothermal temperature. The photocatalytic activity increased first with the increasing hydrothermal temperature from 80 °C to 110 °C and then decreased with the further increasing hydrothermal temperature from 110 °C to 140 °C, indicating the presence of an optimal hydrothermal temperature. This nonlinear dependence between photocatalytic activity and hydrothermal temperature can be attributed to the influence of competition between the area and thickness of ZnO nanosheets at different hydrothermal temperatures. It is well known that the excellent photodegradation performance of nanosheet arrays is mainly due to their large specific area and atomic thickness [16]. The larger the area, the more active sites can be provided. The thinner the thickness, the faster the charge transfer and the lower the recombination rate. As mentioned above, the area and thickness of the nanosheet gradually increased with the increasing hydrothermal temperature. When the hydrothermal temperature increased from 80 °C to 110 °C, the positive influence brought by the increase in the area was a major factor and led to the enhancement of photocatalytic performance. When the hydrothermal temperature was further increased from 110 °C to 140 °C, the negative effect of the increasing thickness greatly reduced the charge transfer and, thus, ultimately decreased the photocatalytic activity of ZnO nanosheet arrays.

To quantitatively understand the hydrothermal temperature effect on the reaction kinetics of the RhB degradation, the pseudo-first-order kinetic fitting plots of as-fabricated supported nanosheet arrays grown at various hydrothermal temperatures are shown in Figure 6b and the corresponding photocatalytic rate constants, are illustrated in the inset of Figure 6b. It was clearly observed that with the increasing hydrothermal temperature, the photodegradation rates of the fabricated supported nanosheet arrays grown at various temperatures first increased and then decreased, and the sample at 110 °C exhibited the highest photodegradation activity with the rate constant k = 0.00935 min^−1^, which is almost four times more efficient than that of ZnO nanosheet arrays grown at 140 °C (0.00276 min^−1^).

**Figure 7:**
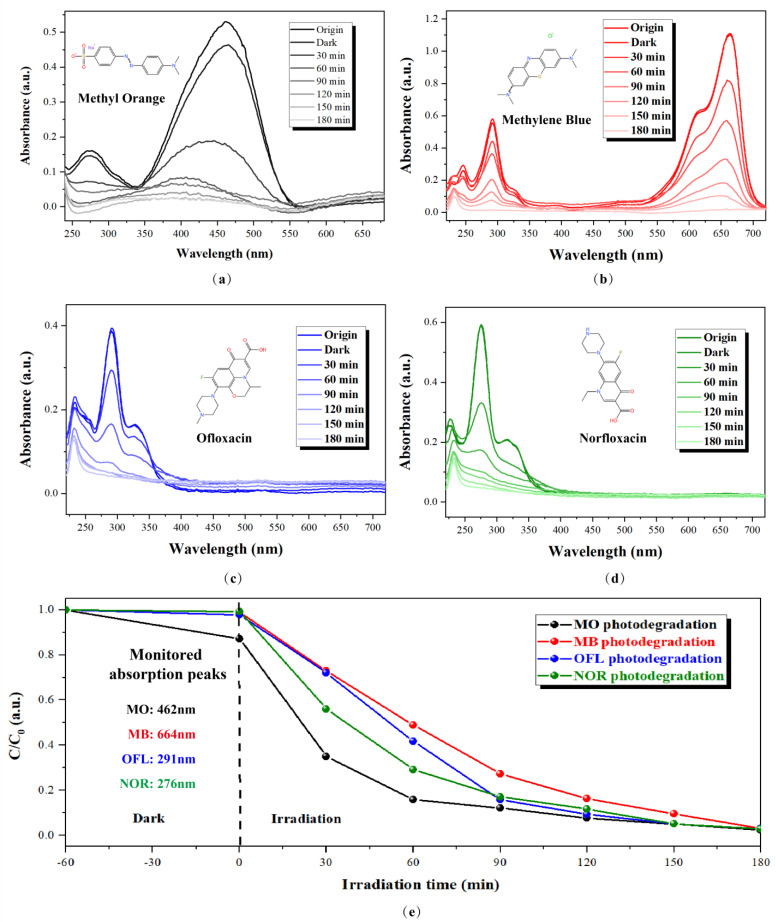
Changes in the UV-Vis absorption spectrum of photodegraded various pollutants with supported nanosheet arrays grown at 110 °C as a function of irradiation time (**a**) MO (**b**) MB (**c**) OFL (**d**) NOR, and (**e**) Pollutants concentration changes as a function of irradiation time.

**Figure 8:**
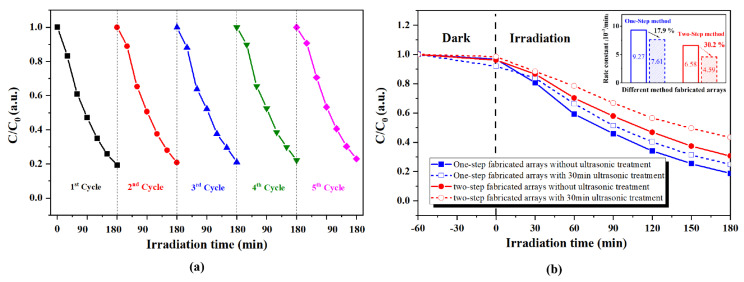
(**a**) Photocatalytic recycling test of supported nanosheet arrays (**b**) Comparison of photocatalytic durability test between one-step and two-step fabricated and supported nanosheet arrays.

In order to verify that fabricated nanosheet arrays can be used in the degradation of various water pollutants, the photodegradation experiments of four different pollutant solutions (10 mg/L) were performed, including MO dye, MB dye, OFL antibiotics, and NOR antibiotics. As is known to all, MO and MB are widely utilized as coloring agents in plastics, painting, cosmetics, paper, leather, and food industries. OFL and NOR are fluoroquinolones antibiotics and are extensively used in agricultural and veterinary treatments. Moreover, as the typical anionic and cationic organic dye, MO and MB differ in their superficial charge. Therefore, testing the photodegradation efficiency of four of these pollutants is helpful to comprehensively understand the degradation ability of as-fabricated ZnO nanosheet arrays for different types of pollutants in water pollution. Figure 7a–d demonstrates the changes in the UV–Vis absorption spectrum of the photodegraded MO, MB, OFL, and NOR solution as a function of irradiation time with supported nanosheet arrays grown at 110 °C. As can be seen from Figure 8, the concentrations of four these solutions were also significantly reduced, and the pollutant solutions were almost completely degraded in 180 min. However, the process of photodegradation is not the same for different pollutants. The degradation of MO was rapid in the first 60 min and then gradually slowed down. As a comparison, the degradation rate of MB was consistent within 180 min. The degradation rates of OFL and NOR antibiotics were in the middle. For instance, after 60 min, 85% MO, 51% MB, 58% OFL, and 71% NOR were degraded. The degradation rates in 60 min followed the order of MO > NOR > OFL > MB. The high degradation efficiency of MO could be attributed to the high photocatalytic activity of the ZnO nanosheet arrays but also to the electrical properties of the dyes. As a typical anionic dye, MO exhibits a negative superficial charge, and the electrostatic attraction between the positive surface charge of the ZnO (0001) surface and anionic dye allowed more MO adsorption onto the surface of the ZnO nanosheet arrays. As a comparison, MB is a typical anionic dye, and its superficial charge is positive. Therefore, MB exhibits less photodegradation activity than MO due to its lack of electrostatic attraction between the dye and ZnO nanosheet arrays. These results indicate that supported nanosheet arrays have great potential for practical and composite water pollution treatment.

In order to clarify the photocatalytic activity of ZnO nanosheet arrays prepared in this work, the degradation of various pollutants was compared with the previous work in Table 2, including cationic, anionic dyes, and fluoroquinolones antibiotics. As indicated in Table 2, compared with the previous work, the improvement of the degradation ability of cationic dyes with the zinc oxide nanosheet structure prepared in this paper is limited, but the degradation abilities of anionic dyes and antibiotics are significantly improved.

As known, photocatalytic cyclical stability and durability are very significant in practical applications besides the photocatalytic performance, especially for supported nanostructured photocatalysts. The cyclical stabilities of as-fabricated ZnO nanosheet arrays for RhB dye photodegradation were investigated by recycling the ZnO nanosheet arrays five times, and the results are shown in Figure 8a. To quantitatively demonstrate the repeatability and stability of the performance of the sample, the average value and standard variance of C/C0 under different irradiation times at five cycles were calculated and listed in Table S1. From Figure 8a and Table S1, it can be seen that no significant loss of activity indicates how supported nanostructured photocatalysts can retain stable recyclability in the process of RhB dye photodegradation. It is worth mentioning that in our experiment, the reused samples only need to be simply washed and dried rather than collected and separated. This further demonstrates the advantages of the ZnO nanosheet arrays prepared in this work for the reuse of photocatalysts.

To investigate the robustness and durability of as-fabricated ZnO nanosheet arrays, destructive simulation experiments were performed using high-energy ultrasonic cleaning (100 W, 30 min). The robustness and durability of fabricated ZnO nanosheet arrays, investigated by the one-step hydrothermal method proposed in this paper and the two-step seed crystal growth method proposed in the literature [29], were compared. Figure 8b shows the photocatalytic activities of two types of ZnO nanosheet arrays before and after 30 mins of ultrasound destructive treatment. As illustrated in Figure 8b, the photocatalytic activity of ZnO nanosheet arrays prepared by the one-step hydrothermal method is slightly higher than that of ZnO nanosheet arrays prepared by the two-step method regardless of ultrasonic destructive treatment prior to or after processing. This may be due to the additional adsorption and photocatalysis of ZnAl LDH. It can further be observed from the inset of Figure 8b that the photocatalytic activity of ZnO nanosheet arrays prepared by the one-step hydrothermal method decreased by 17.7% after ultrasound destructive treatment. As a comparison, the photocatalytic activity of the two-step seed crystal growth hydrothermal fabricated ZnO nanosheet arrays decreased by 30.2% after ultrasound destructive treatment. This result implies that the one-step hydrothermal method is superior to the two-step method in the aspect of photocatalyst robustness and durability. The excellent durability of ZnO nanosheet arrays can be attributed to the direct growth of ZnO nanosheet arrays on the Aluminum substrate without any additional seed layer deposition or surface treatment. Additionally, ZnAl LDH can be formed between the Al substrate and ZnO nanosheet arrays, and this provides good adhesion of the ZnO nanosheet arrays to the substrate [17].

## 4. Conclusions

In summary, large-scale morphologically controlled ZnO nanosheet arrays on aluminum substrates can be successfully fabricated via a one-step hydrothermal process. Due to the presence of the HMTA and Al substrate, $\mathrm{Al}{\left(\mathrm{OH}\right)}_{4}^{-}$ could quickly form and strongly bind to the ZnO (0001) surface, resulting in the growth of vertically aligned nanosheet arrays. By adjusting the hydrothermal temperature, the coverage of the $\mathrm{Al}{\left(\mathrm{OH}\right)}_{4}^{-}$ ions on the (0001) surface planes can be effectively tuned and the area and thickness of as-fabricated nanosheet arrays are eventually controlled. Compared to ZnO nanosheet arrays grown at other hydrothermal temperatures, ZnO nanosheet arrays grown at 110 °C have an optimal surface area and thickness, striking a balance between providing more active sites with the increase in the surface area and reducing the charge transfer with the increase in thickness. The optimized ZnO nanosheet arrays prepared in our work exhibit several attractive features, including efficient photodegradation, facile fabrication, high recyclability, and improved durability. Firstly, the RhB photodegradation efficiency of ZnO nanosheet arrays grown at 110 °C was increased more than four times that of the unoptimized structure. With 10 cm^2^ as-fabricated ZnO nanosheet arrays, the degradation ratio of 10 mg/L, MB, OFL, and NOR is 85%, 51%, 58%, and 71% under UV irradiation (365 nm, 20 mW/cm^2^) for 60 min. All the target pollutant solutions are almost completely degraded under UV irradiation for 180 min. Secondly, ZnO nanosheet arrays were facilely fabricated by one-step hydrothermal methods without any additional seed layer deposition or surface treatment. This will shorten the process flow and reduce the equipment requirements. Thirdly, as a typical nanostructured photocatalyst, ZnO nanosheet arrays can retain stable recyclability, and the reused samples only need to be simply washed and dried rather than collected and separated. This will simplify the post-treatment process of reuse and reduce operating costs, benefitting the scale-up of water pollution photodegradation application. Lastly, due to the direct growth of ZnO nanosheet arrays on the aluminum substrate, as-fabricated ZnO nanosheet arrays exhibit excellent photocatalyst robustness and durability. The as-fabricated ZnO nanosheet arrays not only make the industrial water pollution application sustainable but also have wide applications in solar cells, electronics, optoelectronics, and other fields.

Moreover, further research is needed to elucidate the synergy mechanism between ZnAl LDH and ZnO nanosheet arrays under different growth conditions. In our work, since the ZnAl LDH interlayer between Al and ZnO nanosheet arrays is very thin and covered by dense ZnO nanosheet arrays, it mainly plays the role of adsorption and support and has less a photocatalytic effect. However, when the ZnO nanosheet arrays are incompletely formed or very thin, the effect of the ZnAl LDH interlayer on the photocatalytic activity becomes a major factor. Thus, more research is required to develop a comprehensive understanding of the photocatalytic mechanism of ZnO nanosheet arrays under different growth conditions.

## Figures and Tables

**Table 1:** Surface areas and thickness variations with different hydrothermal temperatures.

Samples Grown at Different Hydrothermal Temperatures	80 °C	95 °C	110 °C	125 °C	140 °C
Surface area (m^2^/g)	15.514	16.244	17.156	19.467	20.362
Nanosheet Thickness (nm)	25.06	32.55	55.00	67.20	105.00

**Table 2:** Comparison of the degradation of various pollutants between this work and previous work.

Pollutants Type	Pollutants	Supported ZnO Photocatalyst	Experimental Conditions	Conclusion	Ref.
Cationic dye	MB	Nest-like ZnO thin films	Solutions: 15 mL 10mg/L, Irradiation: UV 300 W	4 h 86%	[14]
	MB	ZnO nanorod arrays	Solutions: 2 mL 15 μm Irradiation: UV lamp	4 h 60%	[12]
	MB	ZnO grass arrays	Solutions: 60 mL 10 mg/L, Irradiation: UV 300 W	2.5 h 90%	[25]
	RhB	Fe-doped ZnOnanorod arrays	Solutions: ---------------- Irradiation: UV 500 W	3 h 95 %	[26]
	MB RhB	ZnO nanosheet arrays	Solutions: 50 mL 10 mg/L Irradiation: UV 100 W	3 h 98% MB 3 h 81% RhB	This work
Anionic dye	MO	ZnO nanowires arrays	Solutions: 5 mL, 10 μm Irradiation: 50 W xenon lamp	2 h 40%	[10]
	MO	ZnO nanosheet arrays	Solutions: 10 mL 5 μm Irradiation: UV 300 W	4 h 95%	[15]
	MO	ZnO nanosheet arrays	Solutions: 50 mL 10 mg/L, Irradiation: UV 100 W	3 h 99%	This work
Antibiotics	NOR	Cu_2_O/ZnO heterojunction thin films	Solutions: 50 mL 20 mg/L, Irradiation: 250 W xenon lamp	2.5 h 76%	[27]
	OFL	Co-doped ZnO film electrode	Solutions: 50 mL 10 mg/L, Irradiation: 300 W xenon lamp	6 h 86.7%	[28]
	OFL NOR	ZnO nanosheet arrays	Solutions: 50 mL 10 mg/L Irradiation: UV 100 W	3 h 98% OFL 3 h 98% NOR	This work

## Data Availability

The data that support the findings of this study are available from the corresponding author.

## Supplementary Materials

The following supporting information can be downloaded at: https://www.mdpi.com/article/10.3390/nano13030443/s1, Figure S1: Low-magnification top view of the ZnO nanosheet arrays grown at 110 °C (a) the magnification of 2500× (b) the magnification of 5000×; Figure S2: Surface areas analysis of nanosheet (including the aluminum substrates) with different hydrothermal temperatures (a) 80 °C (b) 95 °C (c) 110 °C (d) 125 °C (e) 140 °C (f) thickness variations with different hydrothermal temperatures; Figure S3: Thickness analysis of nanosheet with different hydrothermal temperatures (a) 80 °C (b) 95 °C (c) 110 °C (d) 125 °C (e) 140 °C (f) thickness variations with different hydrothermal temperatures; Figure S4: Variations of XPS spectra with different hydrothermal temperatures (a) the wide scan, (b) the narrow scan of Zn 2p, (c) the narrow scan of Al 2p; Figure S5: Changes of UV–Vis absorption spectra of RhB solution with nanosheet arrays grown at various hydrothermal temperatures as a function of irradiation time (a) 80 °C (b) 95 °C (c) 125 °C (d) 140 °C; Table S1 Average value and standard variance of C/C0 under different irradiation time at five cycles.

## References

[1] Lincho J., Zaleska-Medynska A., Martins R.C., Gomes J. (2022). Nanostructured photocatalysts for the abatement of contaminants by photocatalysis and photocatalytic ozonation: An overview. Sci. Total Environ..

[2] Rajput R.B., Shaikh R., Sawant J., Kale R.B. (2022). Recent developments in ZnO-based heterostructures as photoelectrocatalysts for wastewater treatment: A review. Environ. Adv..

[3] Adnan M.A.M., Julkapli N.M., Hamid S.B.A. (2016). Review on ZnO hybrid photocatalyst: Impact on photocatalytic activities of water pollutant degradation. Rev. Inorg. Chem..

[4] Rupa E.J., Kaliraj L., Abid S., Yang D.-C., Jung S.-K. (2019). Synthesis of a Zinc Oxide Nanoflower Photocatalyst from Sea Buckthorn Fruit for Degradation of Industrial Dyes in Wastewater Treatment. Nanomaterials.

[5] Ye C., Bando Y., Shen G., Golberg D. (2006). Thickness-Dependent Photocatalytic Performance of ZnO Nanoplatelets. J. Phys. Chem. B.

[6] Kumar M., Bhatt V., Kim J., Abhyankar A.C., Chung H.-J., Singh K., Cho Y.B., Yun Y.J., Lim K.S., Yun J.-H. (2021). Holey engineered 2D ZnO-nanosheets architecture for supersensitive ppm level H_2_ gas detection at room temperature. Sens. Actuators B Chem..

[7] Kim K., gyu Choi P., Itoh T., Masuda Y. (2022). Atomic step formation on porous ZnO nanobelts: Remarkable promotion of acetone gas detection up to the parts per trillion level. J. Mater. Chem. A.

[8] Ong C.B., Ng L.Y., Mohammad A.W. (2018). A review of ZnO nanoparticles as solar photocatalysts: Synthesis, mechanisms and applications. Renew. Sustain. Energy Rev..

[9] Geng C., Jiang Y., Yao Y., Meng X., Zapien J.A., Lee C.S., Lifshitz Y., Lee S.T. (2004). Well-Aligned ZnO Nanowire Arrays Fabricated on Silicon Substrates. Adv. Funct. Mater..

[10] Wang G., Li Z., Li M., Feng Y., Li W., Lv S., Liao J. (2018). Synthesizing vertical porous ZnO nanowires arrays on Si/ITO substrate for enhanced photocatalysis. Ceram. Int..

[11] Ma T., Guo M., Zhang M., Zhang Y., Wang X. (2007). Density-controlled hydrothermal growth of well-aligned ZnO nanorod arrays. Nanotechnology.

[12] Di Mauro A., Fragalà M.E., Privitera V., Impellizzeri G. (2017). ZnO for application in photocatalysis: From thin films to nanostructures. Mater. Sci. Semicond. Process..

[13] Wang L., Zheng Y., Li X., Dong W., Tang W., Chen B., Li C., Li X., Zhang T., Xu W. (2011). Nanostructured porous ZnO film with enhanced photocatalytic activity. Thin Solid Films.

[14] Sun F., Qiao X., Tan F., Wang W., Qiu X. (2012). Fabrication and photocatalytic activities of ZnO arrays with different nanostructures. Appl. Surf. Sci..

[15] Chen Y., Zhang L., Ning L., Zhang C., Zhao H., Liu B., Yang H. (2015). Superior photocatalytic activity of porous wurtzite ZnO nanosheets with exposed {001} facets and a charge separation model between polar (001) and (001¯) surfaces. Chem. Eng. J..

[16] Banerjee A., Chattopadhyay S., Kundu A., Sharma R.K., Maiti P., Das S. (2019). Vertically aligned zinc oxide nanosheet for high-performance photocatalysis of water pollutants. Ceram. Int..

[17] Kim K.-H., Kumar B., Lee K.Y., Park H.-K., Lee J.-H., Lee H.H., Jun H., Lee D., Kim S.-W. (2013). Piezoelectric two-dimensional nanosheets/anionic layer heterojunction for efficient direct current power generation. Sci. Rep..

[18] Barreca D., Gasparotto A., Maccato C., Maragno C., Tondello E. (2007). ZnO Nanoplatelets Obtained by Chemical Vapor Deposition, Studied by XPS. Surf. Sci. Spectra.

[19] Ren C., Yang B., Wu M., Xu J., Fu Z., Lv Y., Guo T., Zhao Y., Zhu C. (2010). Synthesis of Ag/ZnO nanorods array with enhanced photocatalytic performance. J. Hazard. Mater..

[20] Murillo G., Leon-Salguero E., Martínez-Alanis P.R., Esteve J., Alvarado-Rivera J., Güell F. (2019). Role of aluminum and HMTA in the hydrothermal synthesis of two-dimensional n-doped ZnO nanosheets. Nano Energy.

[21] Bai Y., Zhao J., Lv Z., Lu K. (2020). Enhanced piezocatalytic performance of ZnO nanosheet microspheres by enriching the surface oxygen vacancies. J. Mater. Sci..

[22] Smith D.Y., Segall B. (1986). Intraband and interband processes in the infrared spectrum of metallic aluminum. Phys. Rev. B.

[23] Liqiang J., Yichun Q., Baiqi W., Shudan L., Baojiang J., Libin Y., Wei F., Honggang F., Jiazhong S. (2006). Review of photoluminescence performance of nano-sized semiconductor materials and its relationships with photocatalytic activity. Sol. Energy Mater. Sol. Cells.

[24] Sameie H., Sabbagh Alvani A.A., Naseri N., Salimi R. (2018). The effect of reduced graphene oxide on photo-catalytic degradation Rhodamine B. Adv. Mater. New Coat..

[25] Wang Z., Ye X., Chen L., Zhang L., Wang Q., Ma L., Hua N. (2020). Photocatalytic properties of ZnO thin film with different morphologies from seed, array to grass. Micro Nano Lett..

[26] Han C., Duan L., Zhao X., Hu Z., Niu Y., Geng W. (2019). Effect of Fe doping on structural and optical properties of ZnO films and nanorods. J. Alloys Compd..

[27] Yu X., Chen H., Ji Q., Chen Y., Wei Y., Zhao N., Yao B. (2021). p-Cu2O/n-ZnO heterojunction thin films with enhanced photoelectrochemical properties and photocatalytic activities for norfloxacin. Chemosphere.

[28] Tian Y., Cheng L., Zhang J. (2018). Fabrication of Co-Doped ZnO Photoanode by Liquid Phase Deposition for Photoelectrocatalytic Degradation of Ofloxacin under Visible Light. J. Electrochem. Soc..

[29] Ridha N.J., Alosfur F.K.M., Jumali M.H.H., Radiman S. (2020). Effect of Al thickness on the structural and ethanol vapor sensing performance of ZnO porous nanostructures prepared by microwave-assisted hydrothermal method. Nanotechnology.

