# Supplementary material for: Morphological Control of Supported ZnO Nanosheet Arrays and Their Application in Photodegradation of Organic Pollutants
Source: Nanomaterials (Basel). 2023 Jan 21;13(3):443. doi: 10.3390/nano13030443 (PMC9919505; doi:10.3390/nano13030443)
Supplement: Supplementary file 1 [file nanomaterials-13-00443-s001.zip › nanomaterials-2110709-supplementary.pdf]

Supplementary materials

Morphological Control of Supported ZnO Nanosheet Arrays and Their Application in Photodegradation of Organic Pollutants

Jun Wang [1,*], Bo Gao [1], Dongliang Liu [1], Lin Cheng [1], Yu Zhang [2], Dingze Lu [1], Huawa Yu [1], Aimin Chen [1], Shun Yuan [1], Kaijia Chen [1] and Shiguang Shang [3]

[1] Shcool of Science, Xi’an Polytechnic University, 19 Jinhua South Road, Xi’an 710048, China
[2] Shcool of Science, Xi’an Jiaotong University, 28 Xianning Road, Xi’an 710049, China
[3] School of Electronic Engineering, Xi’an University of Posts and Telecommunications, 1 Chang’an West St. Xi’an 710121, China;

* Correspondence: wjunxpu@126.com

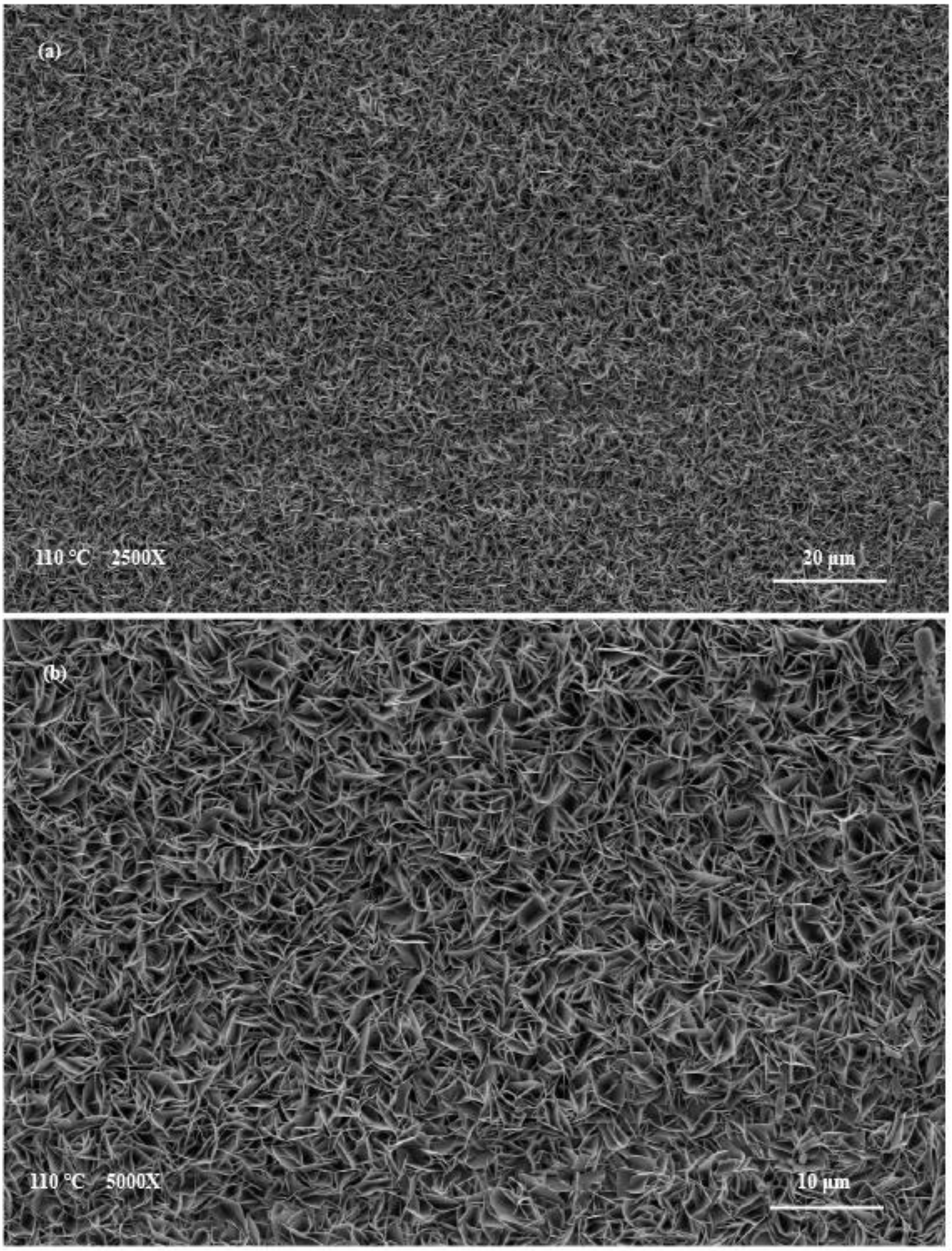

Figure S1 Low-magnification top view of the ZnO nanosheet arrays grown at 110°C (a) the magnification of 2500x (b) the magnification of 5000x

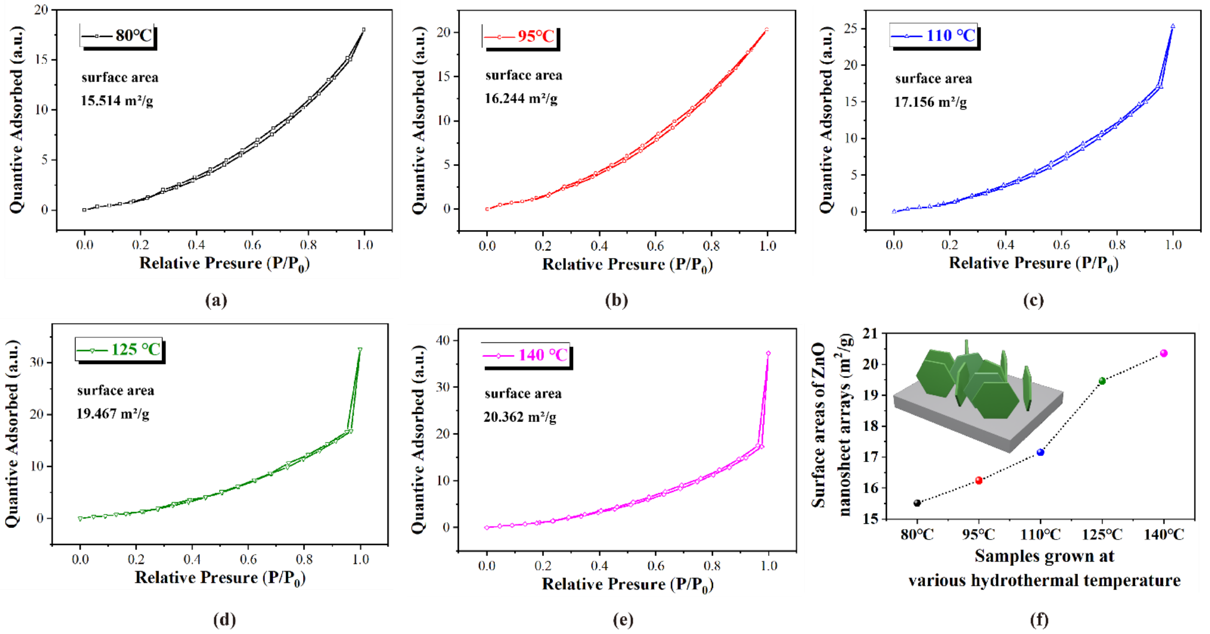

Figure S2 Surface areas analysis of nanosheet (including the aluminum substrates) with different hydrothermal temperatures (a) 80°C (b) 95°C (c) 110°C (d) 125°C (e) 140°C (f) surface areas variations with different hydrothermal temperatures.

It should be mentioned that BET is a block technology. Strictly speaking, these specific surface area obtained by the BET method contains the adsorption information of Al layer, ZnAl LDH layer and ZnO layer. However, due to little changes of the adsorption of the Al layer and ZnAl LDH layer with the increase of temperature, the increase of the specific surface area as-fabricated structure in our work can be mainly attributed to the increase of the surface area of ZnO layer. In fact, it can be seen from the XRD analysis in Figure 2, the diffraction peak corresponding to ZnAl-LDH (003)'s intensity continuously decrease and slightly shifts to a large angle as the hydrothermal temperature increases. The absorbability of ZnAl LDH layer may even reduce with the increase of temperature due to the reduction of the layer spacing.

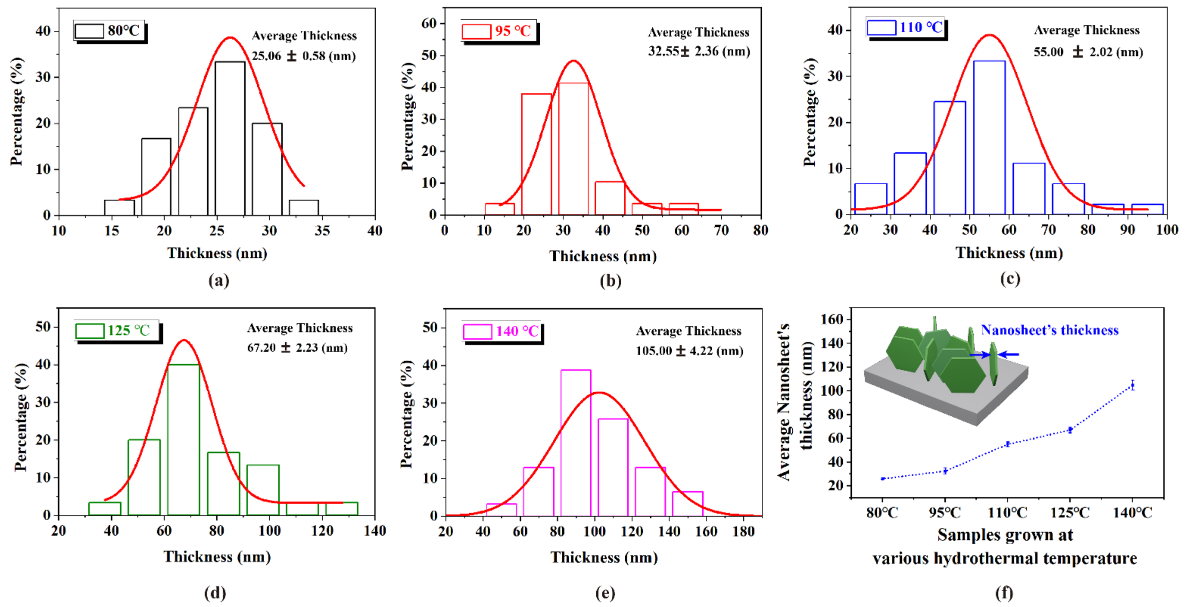

Figure S3 Thickness analysis of nanosheet with different hydrothermal temperatures (a) 80°C (b) 95°C (c) 110°C (d) 125°C (e) 140°C (f) thickness variations with different hydrothermal temperatures

In this work, the thickness variations of the nanosheet were quantitatively revealed by using the thickness statistics and fitting curves methods based SEM results. Vertically aligned ZnO nanosheets grown on the Al substrates and ZnAl LDH interlayers are on the surface of as-fabricated nanostructure. Since the side of nanosheet is exposed to the surface, SEM is suitable for in-situ measurement of ZnO nanosheets' thicknesses. Meanwhile, because a piece of supported ZnO nanosheet arrays contains numerous nanosheets and their growth are not identical, thickness statistics are needed. Considering the nanosheets' vertically arrangement corresponding to SEM analysis, SEM analysis can accommodate a large amount of nanosheets and thus improve the accuracy of statistical results. It is worth noting that there may be minor errors in the thickness variation of the ZnO nanosheets calculated using SEM statistics of the nanosheet sides, as a few nanosheets may have a slight tilt.

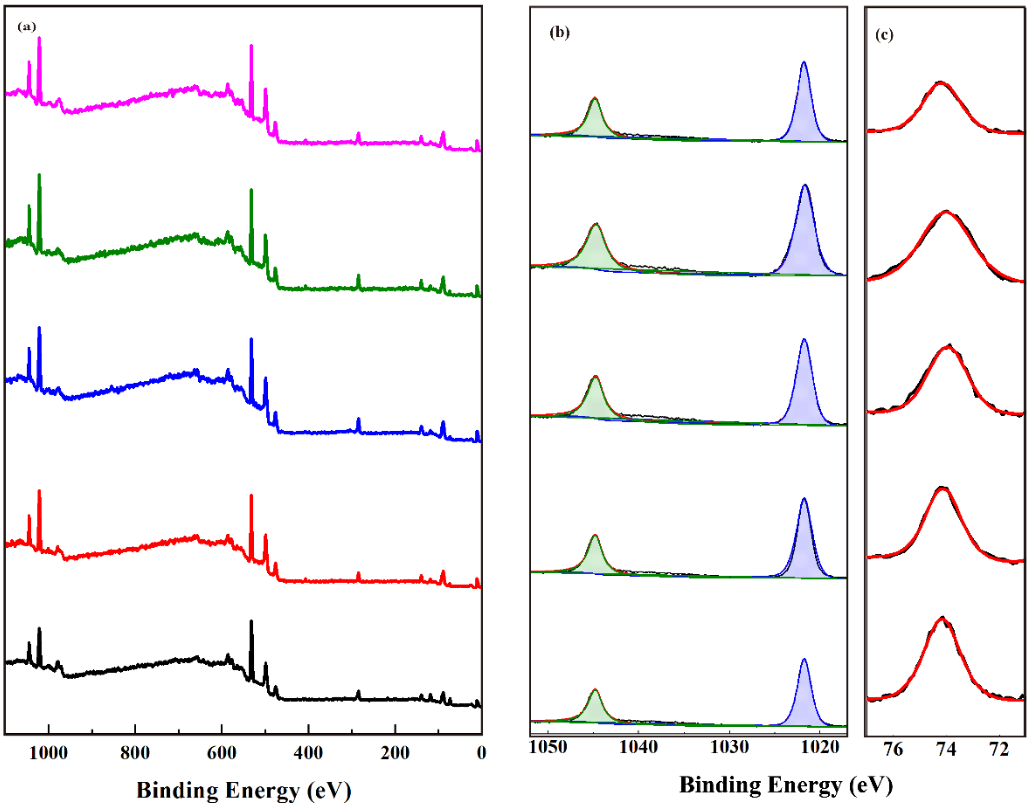

Figure S4 Variations of XPS spectra with different hydrothermal temperatures (a) the wide scan (b) the narrow scan of Zn 2p (c) the narrow scan of Al 2p

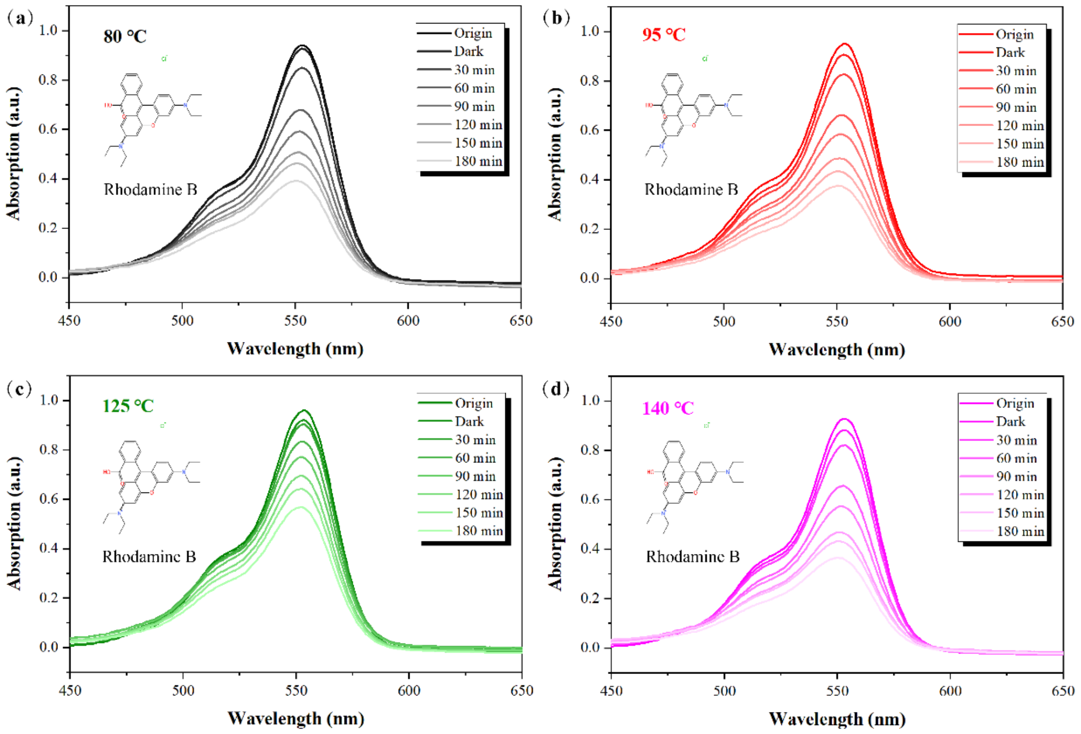

Figure S5 Changes of UV–Vis absorption spectra of RhB solution with nanosheet arrays grown at various hydrothermal temperature as a function of irradiation time (a) 80°C (b) 95°C (c) 125°C (d) 140°C

Table S1 Average value and standard variance of C/C0 under different irradiation time at five cycles

C/C0	1st cycle	2nd cycle	3rd cycle	4th cycle	5th cycle	Average value	Standard variance	Relative error
**30min**	0.83172	0.87855	0.88104	0.89752	0.90652	0.87907	0.028897	3.3%
**60min**	0.60871	0.65241	0.63826	0.65453	0.70605	0.65199	0.035323	5.4%
**90min**	0.47065	0.50577	0.52238	0.52537	0.53337	0.51151	0.024953	4.9%
**120min**	0.34882	0.37519	0.37621	0.38548	0.40571	0.37828	0.020530	5.4%
**150min**	0.25791	0.27934	0.29577	0.29855	0.30241	0.28679	0.018388	6.4%
**180min**	0.19185	0.20714	0.21034	0.22142	0.22947	0.21204	0.014373	6.8%
